# Wars and sweets: microbes, medicines and other moderns in and beyond the(ir) antibiotic era

**DOI:** 10.1136/medhum-2021-012366

**Published:** 2022-08-10

**Authors:** Coll Hutchison

**Affiliations:** Global Health and Development, London School of Hygiene and Tropical Medicine Faculty of Public Health and Policy, London, UK

**Keywords:** COVID-19, history, infectious diseases, public health, metaphor

## Abstract

Once upon a time, many of us moderns dreamt that our future was bright, squeaky clean, germ-free. Now, we increasingly fear that bacterial resistance movements and hordes of viruses are cancelling our medicated performances, and threatening life as many of us have come to know it. In order for our modern antibiotic theatre of war to go on, we pray for salvation through our intensive surveillance of microbes, crusades for more rational antibiotic wars, increased recruitment of resistance fighters and development of antibiotic armaments through greater investment in our medical-industrial-war complex. But not all of us are in favour of the promise of perpetual antimicrobial wars, no matter how careful or rational their proponents aspire to be. An increasing vocal and diverse opposition has amassed in academic journals, newspapers and other fields of practice denouncing medicalisation and pharamceuticalisation of our daily lives, as well as our modern medicine as overly militaristic. In this paper, rather than simply rehearsing many of these well-made and meaning debates to convert you to yet another cause, I enrol them in redescriptions of our modern medical performances in the hope of awakening you from your aseptic dream. What follows is my invitation for you to re-enact our mythic antibiotic era in all its martial g(l)ory. I promise that it will bring you no physically harm, yet I can't promise it will leave your beliefs unscathed, as you follow its playful redescription of how our objective scientific descriptions, clinical prescriptions, economic strategies, political mandates and military orders, not to mention our warspeak, have always been deeply entangled with triumphs and devastations of The(ir) Great anti-Microbial Wars (aka our antibiotic era).

## Introducing you, the(ir) actor

Thank you for joining us for this other—than—(post)modern play. Before our performance begins, first a little backstory to help set (y)our stage, introduce our fellow actors and get you in the(ir) right mood.

The script you are likely familiar with, goes something like this. For much of the 20th century, some of us prophesised an aseptic future that we would realise through medicated acts of antimicrobial war. We cast ourselves as heroic scientists, doctors and big pharma equipped with miraculous medicines, facing terrible but vulnerable microbes, invading unsuspecting, innocent patients. However, come the 21st century, microbes are once again disrupting our modern medicated performances. This has been a surprising plot twist for many. For others, however, it was just a matter of time before bacterial resistance movements and viral hordes disrupted our modern medical performances and cancelled our long prophesised aseptic future. Some of us pleaded for stricter adherence to our (pre)script(ion)s and roles. Many of us did or could not act along, and increasingly fear the imminence of our postantibiotic apocalypse (Podolsky 2018; Podolsky and Lie 2016).

To avert this catastrophe, we must all urgently (re)act to unlearn the roles and scripts we have followed, and actively rewrite them so everyone is aware that we are increasingly confronted by: terrifying resistant superbugs, armed with useless or helpless antimicrobial weapons; besieged by overdemanding (im)patients and overfed swine, irrationally guzzling antibiotics like sweets; over precautious, antimicrobial warmongering doctors (Ruddy 2012; McGrath 2014); and pill pushing pharmaceutical dealers as our fragile lines of defence. We have also recast vets and farmers, as well as their animals and plants in our image, as antibiotic abusers and overconsumers (Kahn 2016). Now we issue martial prescriptions to expand our medical armaments, rationalise their deployment and conscript those of us who have survived onslaught from bacterial resistance movements. All of this, so we can intensify our wars, save our antibiotics and secure our modern medicine (Davies, Grant, and Catchpole 2013; Kahn 2016; Chan 2012; Hall, McDonnell, and O’Neill 2018).

Apologies, perhaps we have gotten ahead of ourselves and become too prescriptive; assuming you know your role and rank or at least, that you follow it without question, without resistance. Do you recognise yourself among our modern medicated cast? If so, can you recollect when you were recruited and assigned your role? Or perhaps you consider yourself outside, excluded from our play: a critical academic dissident, a conscientious probiotic objector or an other-than-Modern? Or maybe you have never entertained or knowingly acted in our modern theatre of war? Maybe you are totally unfamiliar with our antibiotic proscriptions and warmongering? Microbes are not—(post)modern—like you after all. Maybe all this is war is mere metaphorical warspeak?!

Nevertheless, here you are. Maybe for entertainment’s sake or perhaps something more seriously silly, a sneaking suspicion, a gut feeling that you are sometimes following scripts not of your own writing. Cast in roles not fully of your choosing. Acting out scenes on sets and stages, often beyond your comprehension and control. Not that there is anything intrinsically wrong with following scripts or playing along with your roles. After all, your body breathes and digests for you, none of which depends on your conscious awareness. Similarly, most of the food you eat, the medicines you take, the language(s) you speak and the work you do, also depend on multitudes, including microbes and other other-than-Moderns, as well as those who lived, cooked, spoke and laboured before and for you. Sure, your doubts over any of these may lead to bouts of anxiety, despair, rage and even dis-ease, as you question their meaning and consequences they hold for you: microbe, medicine, (post)modern. But as you reflect on who, how and what you put your faith in, these moments might also sometimes(pace) leave you with room—no matter how small—for improvisation, experimentation and variation with the lines you speak, choreographies you follow, ways you act, roles you take on, fellow cast you acknowledge, sets and stages you perform on. For instance, how has it transpired that, despite intensified bacterial resistance movements, repeated viral invasions and decades of critical and damning reviews of our modern medical scripts (Latour 1988; Walker 2020; Dixon et al. 2021; Servitje 2021; Irwin 2020; Sontag 2001), performances and casts (Bud 2007b; Venkat 2021; Podolsky 2014; Kirchhelle 2020; Chandler, Hutchinson, and Hutchison 2016; Koch 2013), many of us still cry for our theatres of war to continue inextricably onward?

Perhaps if we re-enact our modern medical play, we might be gifted with some insights as to how the ‘great’ performances of our science, war, economics and (geo)politics over the last 150+ years are also central to the triumphs and devastations of the(ir) antibiotic era. Maybe, we will even be graced with glimmers of hope at the ends of our modern medicine. So, without further ado reader, please step out of character, put aside your scripts and imagine yourself, watching—or perhaps better said, acting in—a more-than-medical sci-fi play, in which the Earth or Nature is your stage and you are among the lead actors (Danowski and Viveiros de Castro 2017; Latour 1988): scientists, industrialists, military, politicians, medics, patients, microbes, medicines. Please do not take all that follows too literally1: after all, this is not intended as a clinical prescription, objective scientific description nor a military command. Rather it is The Chronicles of the(ir) Great anti-Microbial Wars (Koshland 1992; Lettau and Lettau 2000), two intertwined scientific fabulations: one predominantly (anti)bacterial and its currently unfolding sequel, mostly, but certainly not exclusively, (anti)viral.

## The Chronicles of the(ir) Great anti-Microbial Wars

Although our earliest recorded sightings of microlife go back to the 17th century (Lane 2015), some of us believe that *The Great anti-Microbial Wars* (GMW) extend as far back as the emergence of (hu)man(ity), if not the very dawn of life and death on Earth, such that war may indeed constitute our state of Nature (Dodd 2001; Fuentes 2021). While debates continue to rage in a handful of scholarly circles as to the exact date and origins of our GMWs, our germ aficionado Worboys (2000), not a known war proponent, as his surname might suggest and Tomes (1999), who has written a book or two on Moderns and their germs among others (Bleakley 2017; Servitje 2021; Armstrong 2017), point to our late 19th century as the period when we finally divined the true nature(s) of our dis-ease.

### Religious wars (B.C.–1880s): germs of transcendence and our gospel

Since the *Corpus Hippocraticum*, and assuredly before, we long speculated on the invisible origins of our ills. Some among us advocated for an animacular theory, which proposed that the unseen protagonists of our illnesses were little animals (animalcules). Others suggested that they were the coming together of spontaneous non-living substances (morbid spontaneity), imbalances in bodily fluids (humours), bad air (miasma), poisons (viruses) or plant like seeds (germs) (Barnes 2006; DeLacy 2016; Worboys 2000; Tomes 1999; Nutton 1983; Latour 1988). However, it was not until we retreated and enclosed ourselves in our laboratories, taking with us our domesticated animals, soil and samples of our own and others’ bodies, that we finally witnessed the invisible origins of our ills. Armed with microscopes and petri dishes, we isolated, counted and classified a bewildering abundance and diversity of microscopic actors—proto-animals (protists), small staffs (bacteria), poisons (viruses), sponges (fungi)—which we collectively baptised as germs and immortalised in gospel as our mortal enemies (Tomes 1999). However, for many, what we initially observed in our laboratories was not sufficient proof that germs resided and acted on our farms, in our milk, kitchens and bodies. In order to convert more to our gospel, we brought our laboratories to our farms and hospitals. There, we observed and experimented on ourselves, our cows, rabbits and others alike, meticulously recording what we did, what germs were present or absent, the health of the those we inoculated and our abilities to eliminate them in those we infected. With these revelations, faith in our laboratories as our most holy sites to divine and combat the invisible nature of our dis-ease grew, and with it our dreams of aseptic transcendence and medical progress (Latour 1988).

### Colonial wars (1870s–1930s): germs of dis-ease and their total eradication

As more disciples flocked to our gospel, we established new disciplines (eg, bacteriology) (Worboys 2000), commandments and crusades (Tomes 1999), as well as expanded and transformed already established ones, such as hygiene and sanitation. However, beyond our laboratories, journals and immediate medical and veterinary practices, our gospel remained mostly unknown. The conversion of hygienists to our gospel, with their totalitarian aseptic ambitions and societal reach, helped us transform germs’ relative obscurity into our all-pervasive enemy, ever lurking unseen in our bodies, within our homes and on our streets (Tomes 1999; Rogaski 2014; Latour 1988).

Although we continued to preach that dirt and poor hygiene were a reservoir for our dis-eases, further exegesis of our gospel revealed that germs’ very existence was a constant threat to the maintenance of the favourable order for the filthy rich in our societies (Barnes 2006). So long as our poor, non-Europeans, animals and plants, continued to live out their backward, unscientific and unhygienic lives, we would never truly be free from our germs (Weindling 1993; Latour 1988; Sangodeyi 2014). Thus, our most ardent disciples, those among us who had already attained a state of antiseptic consciousness, believed that it was our moral duty to awaken all those who still lacked the miracles of our modern lavatory—apologies, laboratory—enlightened reason to lead them beyond our dark—and dirty—ages. Therefore, we sought to extend faith in our gospel and commandments of isolate, identify and eliminate to the slumbering bodies and environments of our masses (Kupferberg 2001). With teachings from our gospel, we educated those less fortunate in the wrongness of their ways, to reject practices from their daily lives as culturally backward and follow our scientific commandments and order(s). Some of our more zealous disciples took this further still, dreaming of germs’ total eradication, freeing ourselves finally once and for from our dis-ease (Stepan 2013). To those of us with such privileged vision, we believed our future to be aseptic, germ-free and achievable only through our modern theatre of war. Thus, we began to amass our antigerm armaments and declared The Great Anti-Microbials Wars.

## The(ir) Antibiotic era (1910–present): germs of our modern world order

### Act I. Our aseptic dream: bacterial cleansing for our germ-free future

#### Total world wars (1900s–1947): germs of fascism and mass antibiotic mobilisation

**Figure 1:**
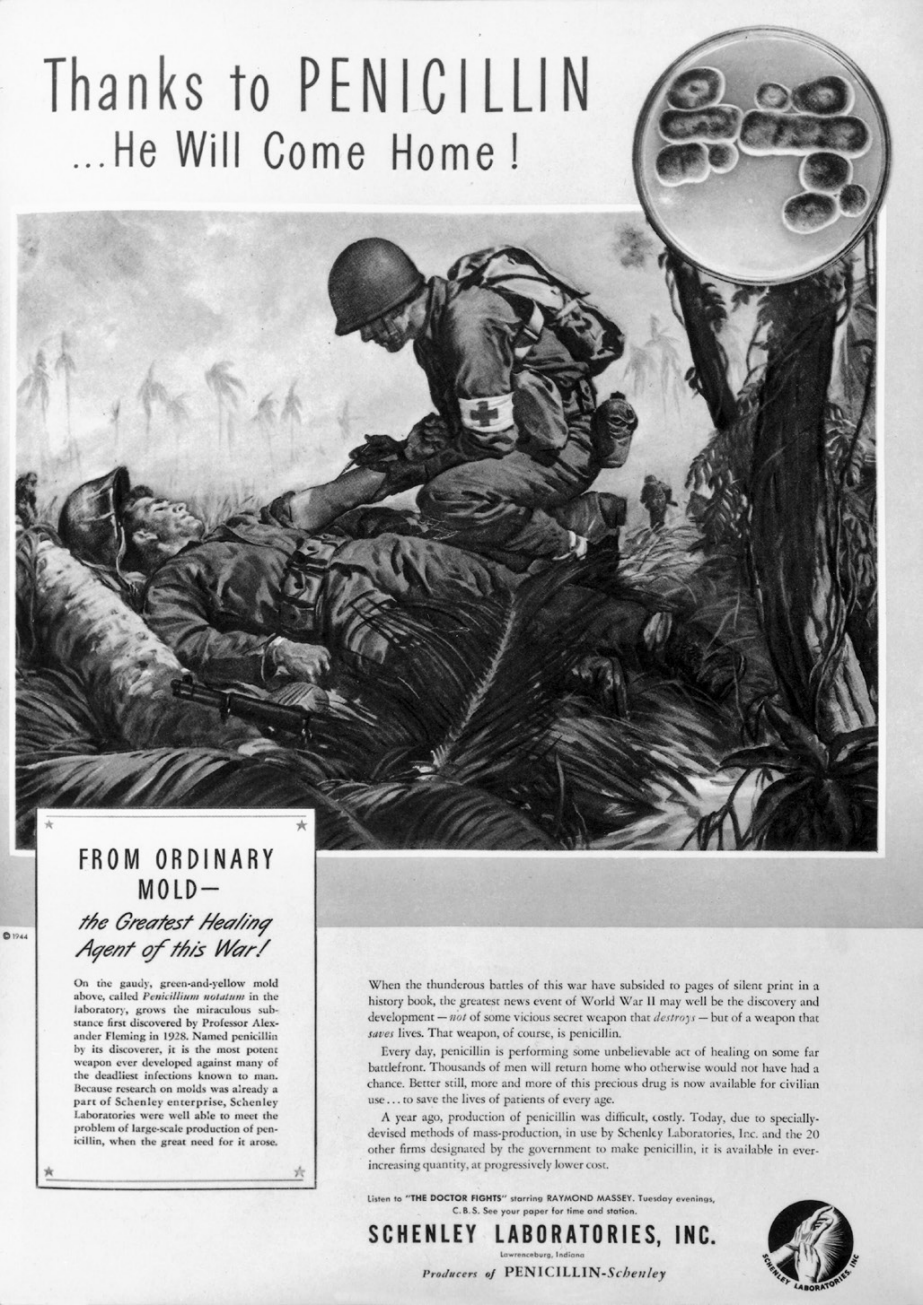
Advertisement for penicillin production from Life Magazine. Science Museum, London. Attribution 4.0 International (CC BY 4.0).

Blessed are our Modern manufacturers of hygiene, medical and veterinary products, who were quick to join our Crusades of Purification against germs. In our advertisements, we consecrated our gospel as central to living our modern daily lives; praying on our masses emerging germ panic to boost demand and sales of our antigerm arsenal (Tebbe-Grossman and Gardner 2011). Mercury salts were one of our earliest weapons capable of eradicating germs. However, we swiftly abandoned them, following revelations about their wider toxicity. Arsenicals, like Salvasarn, and quantary ammonium compounds (QACs) followed, proving highly effective germ killers (Landecker 2018; Lesch 2007). However, it was our serendipitous discovery of synthetic sulphonamides, particularly Prontosil, and an accidental encounter with a fungal penicillin mould (see figure 1), that many of us believed was pivotal in turning the tide in our struggle during the GMWs. These weapons arrived in the shadow of our World Wars, just as our antisepticonsciousness was peaking (Tomes 2000).

World War II (WWII) was an essential proving ground for our antigerm weaponry. The US’s War Production Board succeeded for the first time in the mass industrial production of antigerm arms, and our troops served as the experimental grounds for testing their administration and efficacy (Quinn 2013). Such was the magnitude of injury and mortality suffered by our troops, that we even filtered their urine to ensure every ounce of our precious antigerm weapons were sent to do battle. Antibiotic shots saved our troops lives, so they could continue to fight our enemies on the(ir) frontlines (Bud 2007b)—all the while producing (anti)bacterial evidence of the possibility of our aseptic dreams. A dream mirrored in our nightmares of our atom bombs nu-clear(ly) insured extinction and Nazis’ aseptic nationalism.

#### Civil Wars (1947–1960s): germs of modernisation and antibiotic quick fixes

Over the subsequent decades, our academic and corporate scientists alike scoured our Earth for new weapons to combat, eradicate and profit off germs, sampling everything from mouldy watermelons to the soils from our colonial frontiers (Quinn 2009). We isolated and drafted any weapons with novel offensive properties to fight off an ever-greater range of bacterial invaders (Podolsky *et al.* 2015). Marshalled under the banner of antibiotics (Waksman 1947), we subdivided them into families, classes and generations, dependent on when we discovered them and the methods they deployed to kill (bacteriacide) or disrupt particular bacterial invasions (bacteriostatic). This, in addition to their ability to target specific groups of bacteria (narrow spectrum) or as weapons of mass bacterial destruction (wide spectrum).

Antibiotics conscripted to fight in our GMWs were not limited to combating existent invaders. Rather they were also mobilised in pre-emptive (prophylactic) strategies to combat risks of future bacterial invasions postsurgery (Vats, Nagpal, and Moorthy 2009; Bud 2007b). We were not, however, the only sites upon which we deployed them to war. Our antibiotics increasingly came to the aid of many of our ill, stressed and dying domesticated animals and plants, which we co-existed and depended upon (Nayiga *et al.* 2020; Urapeepathanapong, de Lima Hutchison, and Chuengsatiansup 2022), as well as allegedly providing us with means to accelerate their growth (Kirchhelle 2018). Some of us even dreamt of antibiotics' potential to promote growth and alleviate suffering among those of us under and malnourished (Podolsky 2017). So lethal and efficient were our antibiotics in rendering germs a mere technical matter, no longer worthy of our fear and moral judgement (Bud 2007b), that we heralded them as ‘wonder drugs’ and ‘magic bullets’ (Amyes 2003).

Our antibiotic weapons were central to founding our modern medicine, agriculture and military (Bud 1998; Kirchhelle 2020; Podolsky et al. 2015; Bud 2011; Landas 2020), and modernisation of our very form of life through helping us: shift our hygiene practices in hospitals and farms (Gradmann 2018); increase our—demands for—efficiency, speed and scale of animal production and medical practices (Kirchhelle 2020; Bud 2006); empower our (im)patients, freeing us from illness and dispelling our dis-ease (Bud 2007a); reduce our need and calls for convalesce (Macfarlane and Worboys 2008); increase modern pharmaceuticals’ and other industries’ profits (Podolsky 2014; Quinn 2013) and ensure we emerged victorious in our wars (Bud 2007b; Landas 2020). All that was required was for us to pop the right pill. So, we developed and marketed a range of tailored antibiotic weaponry to target different aspects of our daily battles for germ-free bodies and environments (Tebbe-Grossman and Gardner 2011), including: soaps, detergents, toothpastes, livestock feed and even attempts at germ-eradicating sweets (candettes), ice-cream and lipstick (Bud 2007b). As antibiotic wars came to be infrastructural to almost every aspect of our daily lives (Chandler 2019), some of us went as far as to prophesise that the time was approaching when our bacterial invaders would become a matter of our past, and our dream of a well-ordered, rational and enlightened society free of germs would finally become our reality (Burnet and White 1972; Podolsky 2014).

#### Cold proxy wars (1960s–1990): germs of communism and antibiotic development

As aseptic peace increasingly reigned over our homelands, we attempted to spread our gospel and dreams further overseas to others’ lands and nations. We waged wars supporting and arming other governments and non-state allies: ideological wars; trade wars; wars on drugs, communism, cancer, hunger, smallpox, malaria, tuberculosis and against many of our other dis-eases. We sought to win over allies to our modern aseptic dreams and fight for (neo)liberal democracy through sharing and providing access to the martial might of our militaries, medicine, agriculture, science and industries. All the while, we exported antibiotics and their means of industrial production as ammunition for our proxy wars (Kehr and Condrau 2016; Santesmases 2018; Bud 2011; Kirchhelle 2018; Landas 2020).

Our aseptic dream was not solely, however, about eliminating the germs of our bodily dis-ease; rather, it was part of our grander (inter)national geopolitical ambitions (Callahan 2017), targeted at eradicating our global ills; communists (Thompson 2020; Ivie 1999), for instance, who threatened the growth and aseptic aspirations of our national and corporate bodies (Fishel 2017). Our pursuit of the—global—end of infectious dis-ease was part of our broader politics of containment against the spread and expansions of our enemies, as well as to prevent our Cold War heating up into a hot mess (Parshley and McDermott 2021). With the fall of the Berlin Wall, the Iron Curtain and the fracturing of the USSR, we declared victory of our geopolitical wars against spread and infestations of communism, and over germs themselves. So strong was our conviction of total victory, that we declared that with our Western liberal democracy, we had reached the end of political (Fukuyama 1989) and natural history. We had finally materialised our aseptic dream; the future threat of microbial invaders was ‘dull’ if not over for us (Bud 2007b; Burnet and White 1972; Podolsky 2014).

### Act II. Microbes’ nightmare: resistance to their imminent aseptic apocalypse

Long before we dreamed our aseptic dream, before our primate ancestors first appeared in the(ir) Eocene, microbial-kind manufactured and exchanged minute molecules in relatively low concentrations. Many of these were signalling agents, codes they used to communicate with their kin and allies to organise their communities into biofilms or along different divisions of labour, initiate their growth and colonisation of new habitats, scavenge for foods and increase their motility. When at higher concentrations in particular forms or combinations (Davies 2006), these molecules also formed and initiated the deployment of a diverse array of communication, defence and weapons systems so that microbial communities could outcompete, repel, predate, poison, coerce and even wage war on rivals and foes (Schlatter and Kinkel 2014; Granato, Meiller-Legrand, and Foster 2019; Linares et al. 2006). Naturally—or rather culturally—microbes also developed their own immunity and means to evade, tolerate or neutralise—‘resist’—effects of such signalling molecules and weapons, so as to defend themselves from attacks and mitigate collateral damage (Chassaing and Cascales 2018).

Microbes’ state of nature persisted for billions of years. But with plants, insects and animals' emergence and our later discovery—accidental or otherwise—of substances that harnessed the diverse powers of intermicrobial signalling (Allen et al. 2010; Clardy, Fischbach, and Currie 2009), the terrains of microbial wars dramatically shifted (Granato, Meiller-Legrand, and Foster 2019). Initially, premodern microbial wars were relatively localised, small in scale and intensity compared with our GMWs to come (Waglechner and Wright 2017; Aminov 2010). With the founding of our modern gospel of the germ and conscription of microbes’ molecules into our daily lives, we finally mustered the audacity to declare a total war to end all microbial wars.

#### Antibiotic bullets and bombs (1910–1990s): modernising resistance movements

Prontosil was one of the first antibiotic weapons we deployed to reap havoc on bacterial-kind (Lesch 2007). While many were left unscathed, Prontosil behaved more like bombs than magic bullets when it came to streptococci; who were particularly sensitive to such attacks, often irrespective of where they resided in or on our bodies. Some streptococci evaded or repelled our early antibiotic attacks, but with the advent of WWII, bacteria entered skirmishes and wars of previously unfathomable scales of devastation. Legions met their end while attempting to colonise our wounds, while others perished trying to establish themselves in our lungs and guts, as well as our barracks, kitchens and hospitals. Increasingly, many other microbes began to innovate—through chance mutations—diverse means of resisting our rapid mass deployment of Prontosil, penicillin and QACs into our bodies and environments (Landecker 2018; Podolsky 2018). Those that did so, survived to pass their tales of resistance on to their future generations. While many bacteria inherited their resistance tactics through vertical forms of communication, others also communicated horizontally (Summer 2008), sharing and exchanging genetic resistant codes between their bacterial tribes, families and species (Danchin 2016). One of these, *Staphylococcus aureus*, was an important actor in some of the earliest resistance movements against penicillin during and after WWII (Vento et al. 2013). We later breiefly thwarted *S. aureus* when we developed and marketed methicillin and vancomycin as anti-*Staphylococcus* weapons (McGraw 1974; Bud 2007b). However, bacteria swiftly innovated novel forms of resistance to vancomycin and methicillin.

During the 50s, 60s and 70s, as our expanding antibiotic armamentarium became an increasingly central pillar to our medical and agricultural practices, bacterial resistance movements also spread, gaining myriad new recruits across our Earth (Podolsky 2018; Bud 2006; Kirchhelle 2020), and developing multiple concurrent defensive tactics in order to survive and proliferate; we christened these multi, extreme and pan drug-resistant movements (Paterson and Doi 2007; Falagas and Karageorgopoulos 2008). Our increased global connectivity, through travel, trade and consumption enabled by our motor, ship and airways (Dancer 2013), increased our movements and bacterial invasions and colonisation into ever new territories; their resistance movements did not respect our national borders, nor bothered with passports or visas.

Not all of us, however, proved equally hospitable bacterial environments. Bacteria after all, like us can be choosey: they have their preferences (Brown and Kelly 2014; Paull et al. 2012). Those of us inhabiting hospitals, war zones (Landecker 2018; Bazzi et al. 2020) and informal urban settlements, were especially welcoming for the likes of *Staphylococcus* and *Acinetobacter baumannii*. Entangled with us, our pigs (Blanchette 2020), shrimps (Hinchliffe, Butcher, and Rahman 2018) and many other animals and plants also provided highly suitable training grounds for their fellow bacterial recruits, following our deployment of antibiotic treatments, mass pre-emptive attacks and growth promoters (Iwu, Korsten, and Okoh 2020; Kirchhelle 2018; Taylor and Reeder 2020; Higuera-Llantén et al. 2018). To bacteria, our aseptic dream was a nightmare, a veritable aseptic apocalypse. Those bacteria that could not resist would eventually cease to exist and so, naturally—culturally—resistance movements emerged as existential necessities to bring into being alternative—modern—futures where bacteria would continue to flourish.

#### Germs of resistance (1907–1990s): our microbial nightmare

We first noticed microbial resistance in our laboratories as early as 1907, when we observed *Trypanosoma* parasites’ susceptibility to synthetic dyes, either drop or stop. It was not until we developed the sulffa drugs like Prontosil in the 1930s, that we recognised the significance of germs’ resistance for our medical practices and research (Creager 2007; Gradmann 2011). Indeed, we observed similar glimmerings of resistance movements immediately following our development of penicillin. Early on, a few of us forewarned of the spectre of bacterial radicalisation and future catastrophe, if anti-resistance campaigns and crusades for the rational use of antibiotics were not appropriately executed (Landecker 2016; Podolsky 2014). However, most of us either had ardent faith in our ability to find new or alter existing weapons to provide an inexhaustible variety of antibiotic re-enforcements, or else bacteria’s apparently small-scale resistance movements lacked the evolutionary capacity and potency to give them an upper hand in our arms race (Podolsky 2018).

The triumphalism of our aseptic dreams, our antibiotic bullets and doctrine of containment over microbial-kind proved short lived. Beginning in the 1980s, microbes appeared to be ‘re-emerging’ and others unfamiliar to us—also referred to as ‘emerging’ infectious diseases—increasingly invaded our hard-won aseptic territories (Bashford 2006). HIV was one of the first global microbial counter-revolutionaries (Tomes 1999). HIV was quickly followed by multidrug-resistant tuberculosis movements, Creutzfeldt-Jakob disease prions, methicillin-resistance *S. aureus*, influenza and Ebola viruses in the 1990s and increasing hysteria of influenza pandemics in 2000s (Tomes 2000), infecting our dreams with germaphobia once again (Tomes 2000; Bud 2007b).

Bacteria and other microbes had outsmarted us (Tel Aviv University 2011; Chou 2014). Our scientific intelligence had largely overlooked bacteria’s ability to share mobile genetic codes horizontally (Summer 2008), extending across what we had believed were species barriers (Danchin 2016). The implications were staggering. We had long been aware that some bacteria were especially fit and that it was encoded in their nature to resist and flourish, while others perished. By the time many of us were awakening to the implications of bacteria’s horizontal resistance tactics, we realised that we were writing ‘…*the history of [our] drug innovation…*’ into their DNA (Landecker 2016) and expanding their resistomes so they could increasingly innovate and share new forms counter micro(bio)political resistance movements (FDA 2021; Paxson 2008; Perry and Wright 2014), and that many of them already had achieved global reach (Shah 2010).

### Act III. Securing our (post)modern dream: germs of our postantibiotic apocalypse

#### Wars of terror (1980s–2000s): germs of insecurity and antibiotic stockpiling

To make matters worse for us, it had been some time since our germaphobia had been a big profit business for our pharmaceutical industries. We had not discovered any truly novel class of antibiotic bullets in decades (O’Neill 2016b), and had shifted our R&D to more lucrative chronic and lifestyle dis-ease markets (Podolsky 2014; Gradmann 2016). Meanwhile, although more numerous than us, our Cold War allies and (post)colonial subjects’ greater economic poverty meant they offered comparatively limited opportunities for generous financial returns. Thus, we condemned them to suffer the violent, inequitable legacies of colonialism, concomitant with our (post)modern globalisation (Hirsch 2021; Hinchliffe 2021). Lacking access to sufficient antibiotic arsenals and medical expertise, while being subject to structural adjustment programmes, national debts, corrupt governments and privatisation of their already underfunded and under-resourced state infrastructures (MacPherson et al. 2021; Manyau et al. 2022), they struggled to materialise our aseptic dream. As such, we increasingly diagnosed their states (Khine Zaw, Bawk, and De Lima Hutchison 2021), the movement of their bodies (McInnes and Lee 2006; Relman, Choffnes, and Mack 2010; Ibrahim 2005), their governments, markets, cultures and behaviours, as a major sources of invasive—bacterial—resistance movements (Collignon et al. 2018). Their very existence was a constant threat to not only to our antibiotic bullets and medicine, but also the security of our nations and realisation of our dream for our global aseptic order (Gürcan 2020; Richardson 2020; Fishel 2017).

Our fears of microbial invasion and the transformation of our dreams into nightmares, reached a new feverish intensity following the 9/11 attacks on our twin towers—described by some as an ‘auto-immunological’ attack on the west (Hui 2017)—and deaths due to letters sent with spores of anthrax insurgents. In an antibiotic re-enactment of WWII, we further militarised our medicine and medicalised our military. Overseas, we sought to root out and eliminate germs of—our—terror, impose sanctions that limited access to medicines, as well as inoculate local populations against infectious fundamentalism (Bell 2012). We were under siege from radicalised germs. During our invasions of Iraq, *Iraqibacter*, with its multiple resistance movements, proliferated in our wounds and followed those of us who survived home (Dewachi 2019). Our governments registered microbes as potential bioweapons, and later as threats to our national and global health security (Caduff 2012). We recruited pharmaceutical companies to supply our national stockpiles and purchased exclusive rights on antibiotics, such as Bayer’s Cipro, which we enlisted as biodefense countermeasures in our war against—microbial—terrorism (Quinn 2009). During these times, our governments invested in antibiotics R&D to bolster our biodefense capabilities, save our medicine and secure our nations. Our national security and private commercial interests were merged, so too were our militaries and medicine in pursuit of our aseptic dream (Cooper 2006; Quinn 2009).

#### Global war capitalism (2010s–present): germs of more-than-human markets and antibiotic futures

It was not till the second decade of our 21st century, that our nationalist awakenings against microbial resistance begun to translate into increasingly urgent calls for our coordinated global action. Initially, these cries were isolated to those of us in global health and other high ranking international positions; many of us were the descendants of those who had first dreamt of the end of microbial invasions. We began by revitalising and escalating our scientific prophecies of apocalypse and warnings of returns to the(ir) *‘dark ages of medicine*’ (Walsh 2014). Holding court among our prophets, Margret Chan, the then Director-General of our WHO, at the ‘Combating Antimicrobial Resistance: Time for Action’ in 2012, declared that if current bacterial resistance movements ‘*…continue unabated, the future is easy to predict…’* Our future would no longer be antibiotic, it would be postantibiotic era; the ‘… *end to modern medicine as we know it*’ (Chan 2012). Microbes would resist and finally, transform our aseptic dream into our nightmare. Without antibiotics, our medicines’ future would be thrown into crisis: enter our global postantibiotic apocalypse.

Under our banner of antimicrobial resistance (AMR) as popularly denoted, we began to rally a global countermovement against microbes. Our calls to arms begun to resound around our world, galvanised through our accelerated use of warspeak and millenarianist cries: we proclaimed that our postantibiotic apocalypse was a catastrophe waiting to happen (Caduff 2008). Some took antimicrobial WAAR (see the World Alliance Against Antibiotic Resistance) more literally than others (Carlet, Rambaud, and Pulcini 2012), with the UK’s research councils forming a ‘*…war cabinet to coordinate research…[to] drive forward important advances in the fight against antimicrobial resistance*’ (MRC 2016). David Cameron, then Prime Minister of the UK, staking out antibiotics as a British invention, set out to further marshal his UK’s role in our global fight back against bacterial resistance. Pivotal to this was his appointment of Jim O’Neil, a celebrated ex-Chief Economist at Goldman Sachs, to chair his UK’s review of microbial resistance movements (O’Neill 2016a), and as an economic strategist, develop the case for our coming (anti)microbial wars.

O’Neil together with other disciples of free market growth economics, diagnosed our impending apocalypse as a devastating market failure (Lezaun and Montgomery 2015; O’Neill 2016a). Our carefree attitude and insatiable consumer demand for health (Bud 2007a)—too much self-interest breeding irrationality—had led to our inefficient guzzling of ‘…*antibiotics like sweets*’ (Ward 2016; Spector 2015). Bacteria’s speed and scale of innovation had surpassed our ability to evolve new antimicrobial countermeasures. Increasing radicalisation of immense cohorts of microbes, contributed to escalating fears of our antibiotic supply pipelines running dry (Spellberg 2011), and threats of AMR increasingly as a ticking time bomb (Wasley, Heal, and Davies 2020; Torjesen 2013).

Simply put, we had not been rational enough, particularly those less educated, poorer, living in low-resourced settings (although many of us vehemently disagreed and refused these evangelical claims: Broom and Doron 2020; Nabirye et al. 2021; MacPherson et al. 2021; Dixon et al. 2021). So, we issued commands to further drill rationality into civilians and medical and veterinary professionals to better protect and stockpile our current and future antibiotic armaments (APUA 2020; Weigel and Morse 2009). We recruited antibiotic guardians (Kesten et al. 2018) and resistance fighters (Longitude Prize 2018), and called for more and more people to sign oaths and pledge their allegiance and funds to our war on bacterial resistance.

Against this foreboding backdrop, Jeremy Hunt, the UK’s Health Secretary, warned of further catastrophic costs of inaction:

[…] the global cost of taking action on AMR is up to $40 billion over 10 years—but this is vastly outstripped by the costs of inaction, which is potentially in the trillions, so we cannot afford not to step up to secure the future of modern medicine (Department of Health 2016)

As a central pillar of our modern lives, massive and rapid economic investment would be required to save our antibiotics and mitigate against our imminent apocalypse (Carlet, Rambaud, and Pulcini 2014). Our recent investments to incentivise the development of new classes of antibiotics (Walsh 2013) had produced weapons that were mostly redundant—none of them could target bacterial resistant extremists (Nielsen et al. 2019), and our pharmaceutical industries were increasingly facing a disincentivising conundrum: how to further motivate antimicrobial innovation, where our desire for more efficient and rational war would potentially limit their future economic returns.

Our solution was to extend our free-market democracy: more and greater government investments, new financial mechanisms to distribute these funds to our pharmaceutical companies and the extension of our markets to microbes. More-than-human market capitalism: we as rational self-interested consumers in pursuit of maximum health; microbes as rational self-interested actors in pursuit of maximised fitness (Amadae 2016). Bacteria’s resistance was not to be overcome, but rather incorporated into the very production of our commercial opportunities and expected financial returns. Bacterias’ constant adaptation to our antimicrobial warfare, through acquisitions and mergers of their resistance tactics was a—genetic—asset for their fitness (Baquero 2004), and for our—future—pharmaceuticals’ profits (Brown and Nettleton 2018). Natural selection and the(ir) market’s invisible hand could finally be united in the name of rational economic and evolutionary efficiency (Jones 2000). Our new antimicrobials would target resistant bacteria and our development of diagnostics would provide us with the simultaneous means for precision kills and greater economic returns. Some of us even pronounced the imminence of an artificial intelligence-assisted antibiotic renaissance (MERCK 2020). Our postantibiotic apocalypse provided us with an unprecedented business opportunity (Helperby 2013; O’Neill 2015), with those of us in the UK aiming to ‘…*corner the Apocalypse market before anyone else*’ (Trémolières et al. 2010; Cooper 2006; Shaviro 2013). In so doing, we would save our medicines, and declare The Great anti-Microbials Wars—a more-than-human war without end—as our form of life (Grove 2019).

## The(ir) vaccine era (2020–present): germs of our new post/modern world (dis)order

Despite increasing attention and escalating fears of bacterial resistance movements, it was viral invasions that initiated our first 21st century total global war against microbes. Our viral prophets had forecasted the imminence of a pandemic for decades. Their studies of the Spanish influenza and other past pandemics concluded that it was not a matter of ‘if’, but ‘when’ the next one would occur (Caduff 2014; MacPhail 2011). Genomic studies later revealed SARS-COV-2 or COVID-19 for short, was lurking in China in late December 2019. Some speculated that they may have spread undetected into Brazil and Europe around the same time or even earlier (Baddal et al. 2021; Carrat et al. 2021; Apolone et al. 2021; Fongaro et al. 2021; Lippi, Henry, and Sanchis-Gomar 2021; Basavaraju et al. 2021), but it was 2020 that was to be the Year of Our Pandemic.

### Act I. Global war (January–March 2020): post/moderns and viral coronisation

China was the first to herald the arrival viral invasions and muster a martial response, locking down Wuhan and other cities in the province of Hubei to contain the spread of the(ir) virus. Non-Chinese moderns, mostly those of us in the Global North and West, as well as other countries who our descendants had invaded and settled, rushed to condemned such measures, proclaiming them to be overly dramatic and militaristic (Sudworth 2020; Xue 2021). Despite China’s strict lockdowns measures, SARS-COV-2 and those with influenza-like symptoms were detected in neighbouring Southeast Asian countries, who responded quickly with track and trace, quarantine and other containment measures. However, our reports of the number of countries infected and the quantity of deaths, continued to increase rapidly. Italy was our first Western nation to initiate a national lockdown. Other countries quickly followed rank with quarantine measures and national, regional and city lockdowns. By March 2020, when our WHO announced that COVID-19 had gone pandemic, it was evident that our societies were no more immune to coronisation than China. The USA and the UK, who ranked ourselves most highly in pandemic preparedness, were among the worst—at least, initially—infected (Abbey et al. 2020). This, however, did not stop our political leaders demanding we remain vigilant in case of viral re-invasion from ‘…*the least protected countries with the most underdeveloped health systems*’ (Sherwood 2020). Thus, we in the USA and the UK were among the first to reverse the microbiologist Dubos’s slogan that to “*Protect ourselves locally means we need to act globally […]*” (Sherwood 2020). Faithful to our gospel and aseptic dream, we feared we would only be secure when our virus was eradicated from every continent and so, we declared a global war (Brives 2020; WHO 2020).

### Act II. National (un)Civil Wars (March 2020 and onwards): lockdown heroes and viral suppression

Our wars against the COVID-19 required a total (de)mobilisation of our societies on scales that many of us could only compared with WWII (Carbonaro 2020; Merrin 2020). Our governments announced national states of emergency and enacted war-time measures to protect our health services, flatten their COVID-19 curves and save their economies; while we awaited development of vaccinations to defend against viral invasions (Stavrianakis and Tessier 2020). We ordered almost everyone to stay home to support our fight on the(ir) virus. Health, police and others deemed essential workers were conscripted to wage wars and potentially sacrifice their lives on our frontlines (Time 2020). Meanwhile, our militaries and private security firms were enlisted as extra recruits and national reserves to track and trace, establish and (wo)man makeshift hospitals, police borders and preserve or enforce our semblance of law and order (Forces Network 2021; G4S 2021).

Lockdowns dramatically disrupted the normalcy of our daily lives. Some of us, whose normal lives were already extreme by most accounts, still managed to take flights and escape to our private islands and bunkers (Hurley 2021; Schiffer 2020), stockpile food, medical necessities and fight over toilet roll (Nature 2020). Meanwhile others of us lost our jobs, were left abandoned and stranded, without food and medical attention (Bennett 2021; Biswas 2021; Bekiempis 2020). Some of us grappled with rising germaphobia and fears of going out or being locked in. Others clashed on social media over social distancing or the existence of COVID-19, while locked in our homes. Some purchased guns and patrolled our streets, proclaiming that they would rather die than closedown the(ir) economy and kill their country (Coppins 2020). Others protested against racism, (neo)colonialism and extinctions that continued to divide our societies, dictate ongoing geopolitical inequities and socioecological catastrophes. Some of us attempted to make time and space to write and reflect on our lives, the conditions of our worlds and fragile existence (Searle, Turnbull, and Lorimer 2021; Stiegler 2020). Many of us hoped to go back to the same old normal, while others dreamed of new ones.

### Act III. New Cold Wars (April 2020): post/modern leaders and viral dissension

A couple of months into coronisation, our UN Security-General appealed for an immediate global ceasefire in order to focus efforts on defeating our virus. Some of us asserted that our wars over territories and resources were getting in the way of fighting the GMWs, which without everyone putting their differences aside and striving for unity and peace, would be lost (UN 2020). While over 200 of our state governments and non-state actors endorsed the ceasefire proposal, a few of our 5 permanent members, including the UK and Russia, refused to sign any agreement that curtailed their counterterrorism measures. The US demanded that any agreement refer to ‘Wuhan virus’ and attempted to block engagement with the(ir) WHO, while China sought to block any criticism of its handling of the(ir) virus and highlighted the US’s lack of international diplomacy and mishandling of its response (Gowan and Pradhan 2020). Already existing ethno-state and regional authoritarian nationalisms, as well as geopolitical tensions were going increasingly viral, including spiralling militarism by the USA, Australia, China, India, Russia, North Korea, Iranian, Saudi Arabian. Our political leaders, Trump America’s ‘War-time President’ (Renfro 2021), Xi Jinping, Prayut Chan-o-cha, Bolsonaro, Modi, Johnson, Aung San Suu Kyi among many others, used COVID-19 as scapegoats for their failings and as a means to garner further support. Rather than pursue ceasefire, peace and a united global war, we waged a global Civil War, with each government fighting and attempting to contain their COVID-19 at subnational and national scales. However, despite our christening of Indian, South African, Brazilian, the quaint Kent (UK) and other viral strains, COVID-19 did not respect our national borders, further undermining our global (war)path to antiviral victory.

### Act IV. War goes viral: pandemic profit(eer)s and vaccine nationalism

But not all of us suffered, or at least not equally. Coronisation accelerated millions into further extremes of impoverishment (Lakner et al. 2021), while intensifying germaphobia and lockdown proved big business for some. Our pharmaceutical companies provided us with diagnostic tests to track and trace COVID-19s, while many of them made hefty profits. Masks and alcohol gel were in high demand too. They served as everyday armour and weapons in our ongoing fight for our aseptic dream. Meanwhile, our governments funded (Cross et al. 2021) Big Pharmas’ development and production of vaccines and stockpiled them as national(ist) defence measures against coronisation and its potentially mortal consequences. While the majority of governments could not afford and source sufficient vaccines for their citizens, Big Pharma was relishing the prospect of reaping hundreds of millions of dollars in profits through sales and technology-transfers (Hassan, Yamey, and Abbasi 2021; Stein 2021; Keestra 2021).

Similarly, coronisation provided opportunities for Big Tech to accelerate the digital(colon)isation of our lives—death inc. (Laura et al. 2020; Kiel 2020). Firms like Google, Facebook, Microsoft, Twitter, Zoom, Amazon and Tik Tok’s stock market values and the personal wealth of their major shareholders increased by tens and hundreds of billions (Torpey, Brockmann, and Hendricks 2021; Elks 2021). Lockdown meant we physically moved less and spent more time and money online. Our physical confinement and distancing measures accelerated digital platforms’ algorithmic translation of our fears, angers, anxieties, lies, joys, meditations and diatribes on microbes, sourdoughs and conspiracies into teraflops of data. Allegedly, our exchange of (mis)information, especially anger and fear, could travel even faster than COVID-19 themselves (Debiec 2020).

Our digital platforms served as virtual battlegrounds for the spread of info viruses and increasing polarisation within our societies. As we increasingly succumbed to infodemics (WHO 2020) of disinformation and conspiracies, we fought counterinsurgencies against such radicalisation through digital sanitation (censorship) campaigns and targeted inoculation of online users with our gospel, dreams and commandments. Alas, many proved immune or allergic. They attacked, rejected and resisted our vaccination campaigns, physical distancing and lockdown orders.

As more of us began to wake from our national COVID-19 free (aka zero COVID-19) dreams, we increasingly begun to open our countries up for followers and leaders of our gospel’s movement. Big Tech helped our governments enforce and police our national borders through tracking and surveilling us. They helped restrict movement of those who opposed, hesitated or could not afford to vaccinate or follow our commandments, as potential viral-and-national threats to our aseptic dream. Coronisation now seemed to be here to stay and all the while, pandemic profiteers, such as Big Tech, Pharma, Finance and other post/modern aspiring—state and market—fundamentalists were making a killing.

Some of us heralded these times as the dawn of a new ‘Vaccine era’, where novel digital and technologies, regulatory mechanisms, supply-consumer chains, funding and private-public collaborations would create more rapid, efficient pandemic preparedness and other infectious disease responses (Quilici 2021; Smith 2016). Some baptised it the(ir) ‘Pandemic era’, and preached that we could only save ourselves if we went beyond our technological fixes and wars to transforming our very relations to—microbial and other—nature (The Lancet Planetary Health 2021).

## Waking up from our aseptic performance

We would like to thank you for joining us in our play and wish you all the best as you to return to your life in other (sub)sets—homes, factories, farms, clubs, offices, laboratories, fields, forests.

Whether or not you continue in character as a (post)modern conscript, we hope you now have a better feel for how: what you ‘dream’, how you ‘act’ and ‘speak’, and who you ‘are-and-are-against’ are already partly scripted, directed and produced through our ongoing (post)modern theatres of war. And that not only are you more than the roles you act out, but you never act alone: microbes, medicines and other (post)moderns are always part of your scripts.

So rather than fearing the end of our modern medicine and continuing to dream an aseptic world, where our antimicrobial wars promise freedom and salvation from germs, consider joining some of us cited in the preceding acts of our GMW. Perhaps together, we can finally put our aseptic dreams to sleep and pursue decoronial and other movements beyond our modern medicated military performances.

## Data Availability

Data sharing is not applicable; as all data was acquired from already published articles, books and media sources, and can be found through following the citations and in the bibliography. No new datasets were generated for the scripting of this play paper.

## Notes

1. Like medical research papers, plays, their scripts and performances draw on, are inspired by and speak to the works of others. This ‘paper’ is no different. It enlists the thought, research and efforts of many others for its creation. It does so as an open invitation to you, reader/actor, to seriously entertain it and its citations to further revaluate your own scripts and performances, as well as hopefully for you to redirect, improvise and add your own.
